# Supplementary material for: Effect of Post–Polyketide Synthase Modification Groups on Property and Activity of Polyene Macrolides
Source: Antibiotics (Basel). 2023 Jan 8;12(1):119. doi: 10.3390/antibiotics12010119 (PMC9854516; doi:10.3390/antibiotics12010119)
Supplement: Supplementary file 1 [file antibiotics-12-00119-s001.zip › antibiotics-2100935-supplementary.pdf]

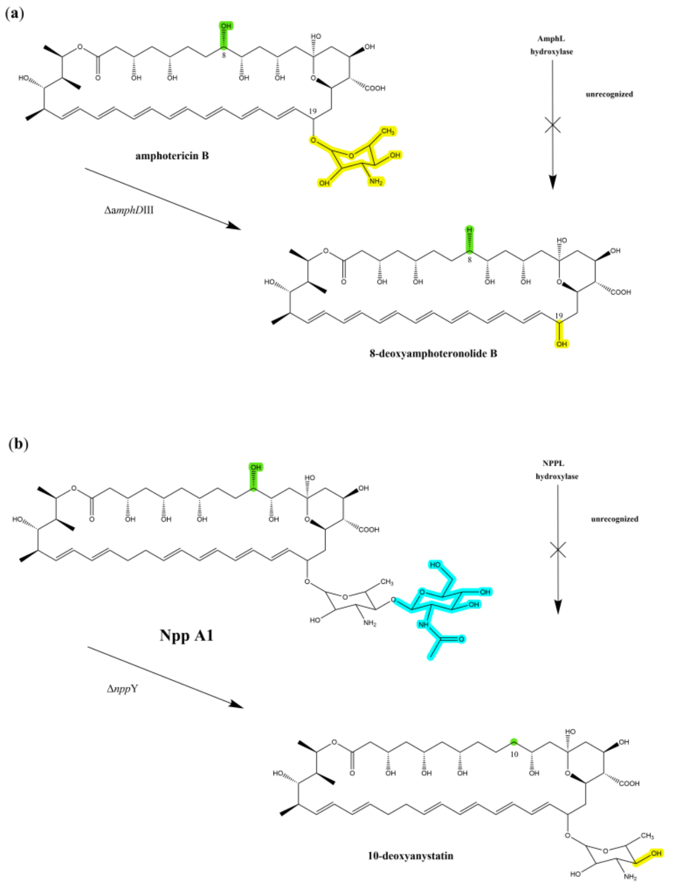

Figure S1. Changes of antibiotics after deletion of specific P450 enzyme gene. (**a**), Blocking *amph*DIII in the amphotericin B biosynthetic gene cluster results in that AmphL cannot recognize the precursor normally, producing 8-deoxyamphoterolide B. The green part shows the structural change after AmphL cannot be recognized normally, and the yellow part shows the structural change after the glycosyl cannot be added normally; (**b**), Blocking *npp*Y in the NPP A_1 biosynthetic gene cluster results in that NppL cannot recognize the precursor normally, producing 10-deoxynystatin. The green part shows the structural changes after NppL cannot be recognized normally, and the yellow and blue parts show the structural changes after the second glycosyl cannot be added normally.
